# Follicular cell-derived thyroid carcinomas harboring novel genetic *BRAF^NON-V600E^* mutations: real-world data obtained using a multigene panel

**DOI:** 10.20945/2359-4292-2024-0067

**Published:** 2024-09-17

**Authors:** Juliana Lima von Ammon, Gabriel Jeferson Rodríguez Machado, Rafael Reis Campos da Matta, Ana Clara Telles, Fabiane Carrijo, Bruno Alexsander França dos Santos, Juliana Cabral Duarte Brandão, Thiago Magalhães da Silva, Fabio Hecht, Gabriel Avela Colozza-Gama, Julia Helena Tezzei, Janete Maria Cerutti, Helton Estrela Ramos

**Affiliations:** 1 Universidade Federal da Bahia Instituto de Ciências da Saúde Programa de Pós-graduação em Processos Interativos de Órgãos e Sistemas Salvador BA Brasil Programa de Pós-graduação em Processos Interativos de Órgãos e Sistemas, Instituto de Ciências da Saúde, Universidade Federal da Bahia, Salvador, BA, Brasil; 2 Universidade Federal da Bahia Instituto de Ciências da Saúde Departamento de Biorregulação Salvador BA Brasil Departamento de Biorregulação, Instituto de Ciências da Saúde, Universidade Federal da Bahia, Salvador, BA, Brasil; 3 Serviço de Patologia do Hospital Aristides Maltez Salvador BA Brasil Serviço de Patologia do Hospital Aristides Maltez, Salvador, BA, Brasil; 4 Universidade Estadual do Sudoeste da Bahia Departamento de Ciências Biológicas Jequié BA Brasil Departamento de Ciências Biológicas, Universidade Estadual do Sudoeste da Bahia, Jequié, BA, Brasil; 5 Universidade Federal do Rio de Janeiro Instituto de Biofísica Carlos Chagas Filho Rio de Janeiro RJ Brasil Instituto de Biofísica Carlos Chagas Filho, Universidade Federal do Rio de Janeiro, Rio de Janeiro, RJ, Brasil; 6 Universidade Federal de São Paulo Departamento de Morfologia e Genética Laboratório de Bases Genéticas de Tumores de Tireoide São Paulo SP Brasil Laboratório de Bases Genéticas de Tumores de Tireoide, Divisão de Genética, Departamento de Morfologia e Genética, Universidade Federal de São Paulo, São Paulo, SP, Brasil

**Keywords:** Thyroid cancer, papillary thyroid carcinoma, *BRAF* ^V600E^, next-generation sequencing, mutation

## Abstract

**Objectives:**

To assess the molecular profile of follicular cell-derived thyroid carcinomas (FCDTCs) and correlate the identified mutations with the clinical and pathological features of the affected patients.

**Materials and methods:**

Cross-sectional study of tumor samples from 100 adult patients diagnosed with FCDTC between 2010 and 2019. The patients’ clinical and pathological data were collected. Genomic DNA was extracted from formalin-fixed, paraffin-embedded (FFPE) tumors using the ReliaPrep FFPE gDNA Miniprep System. Genotyping of target genomic regions (*KRAS, NRAS, BRAF, EGFR*, and *PIK3CA*) was performed using the AmpliSeq panel, while sequencing was performed on the iSeq 100 platform.

**Results:**

The patients’ mean age was 39 years. In all, 82% of the tumors were classic papillary thyroid carcinomas. Overall, 54 (54%) tumor samples yielded satisfactory results on next-generation sequencing (NGS), of which 31 harbored mutations. *BRAF* gene mutations were the most frequent, with the *BRAF*^V600E^ mutation present in 10 tumors. Seven tumors had *BRAF*^NON-V600E^ mutations not previously described in FCDTCs (G464E, G464R, G466E, S467L, G469E, G596D, and the T599Ifs*10 deletion) but described in other types of cancer (*i.e.*, skin/melanoma, lung, colorectal, and others). One tumor had a previously reported *BRAF*^A598V^ mutation. *EGFR* gene mutations were found in 16 (29%) and *KRAS* or *NRAS* alterations in 8 (14%) of the 54 tumors analyzed.

**Conclusion:**

We described herein seven non-hotspot/novel variants in the *BRAF* gene, highlighting their potential role in expanding our understanding of FCDTC genetics.

## INTRODUCTION

Follicular cell-derived thyroid carcinomas (FCDTCs) account for approximately 90% of all cases of thyroid cancer; of these, 80%-85% are papillary thyroid carcinomas (PTCs) (1-5). A subgroup of these tumors exhibits genetic heterogeneity with more aggressive variants, making thyroid cancer more invasive and lethal in these cases (3,4).

Understanding the molecular mechanisms in carcinogenesis is essential for an accurate diagnosis and a personalized therapeutic approach. Next-generation sequencing (NGS) is the gold-standard technology for simultaneous analysis of genes of interest, allowing for a better evaluation of cancer and a specialized therapeutic approach (6,7).

Most genetic alterations that lead to thyroid tumorigenesis have been described in genes encoding the effectors of the MAPK and PI3K-AKT pathways, resulting in dysregulation of cell growth and differentiation. The genetic alterations (*i.e.*, pathogenic variants and fusions) affecting these pathways have been mainly identified in the receptor tyrosine kinases *RET* and *NTRK1,* located in the cell membrane, as well as their intracellular signal transducers, such as *BRAF* and *RAS*. These mutations occur in approximately 70% of all cases of PTC (6,8-14).

The most common somatic mutation found in adult thyroid cancer is harbored in the *BRAF* gene, *i.e.*, the *BRAF*^V600E^ mutation (15,16). This variant is highly prevalent in PTCs, with a frequency of approximately 45% among all PTC cases worldwide (1,8,17,18). Although other *BRAF* mutations – known as *BRAF*^NON-V600E^ – have been described in FCDTCs, further studies are needed to better classify and correlate them with the clinical and pathological features of the affected patients.

The NGS analysis has become part of thyroid cancer care, and customized cancer gene panels are now considered cost-effective and an optimal tissue-saving alternative (19). Performing NGS is a multistep process that typically involves sample acquisition and quality control, DNA extraction, library preparation, sequencing, and genomic data generation (20). Several professional societies have published guidelines for NGS use in various tumors, with a focus on analytical validity and quality (20-22).

As new treatment options emerge, tumor analysis may be required more frequently, especially in patients with driver mutations unresponsive to first-line therapies, *e.g.*, surgery and radioiodine in thyroid malignancies. Endocrinologists, oncologists, and pathologists often collaborate closely to obtain specific insights into preanalytical tissue quality requirements, ensuring adequate material for molecular studies and standardizing specimen handling and processing. However, several factors limit the feasibility of tumor genetic profiling studies, including the amount and condition of the material that can be recovered. Few data have been published on the feasibility of NGS-based molecular studies, particularly in a real-world setting and in Latin American or low-income countries.

Based on these considerations, this study aimed to investigate a customized multigene panel for detecting mutations in thyroid cancer oncogenic drivers (*BRAF, KRAS*, *NRAS, EGFR*, and *PI3KCA*) and correlate the obtained results with clinical and pathological characteristics of the affected patients. The patients were adults with FCDTC treated at the largest oncologic reference hospital in the state of Bahia, Brazil, covered by the Unified Health System (SUS). We hope that our experience will be valuable to other institutions worldwide.

## MATERIALS AND METHODS

### Study design

This was a retrospective, cross-sectional, single-center study of tumor samples from 100 non-consecutive adult patients who underwent thyroidectomy for the treatment of FCDTC at *Hospital Aristides Maltez* (AMH) in Salvador, Bahia, Brazil, between January 2010 and December 2019. The patients were identified through a search using the International Classification of Diseases, 10^th^ revision (ICD-10), code (C73), and a review of pathology records. The patients’ formalin-fixed, paraffin-embedded (FFPE) tumor samples were selected for molecular analysis.

Tumor slides, stained with hematoxylin and eosin (HE), were classified by a pathologist according to the World Health Organization Classification of Thyroid Neoplasms (23), and the tumors were staged according to the American Joint Committee on Cancer (AJCC) Cancer Staging Manual, 8th edition (24). Three 10-µm thick histologic sections were obtained from each selected paraffin block, transferred to pre-sterilized (DNase- and RNase-free) 1.5 mL Eppendorf microtubes, and labeled.

Comprehensive chart reviews were performed to collect the patients’ demographics, family history, and information on previous radiation exposure and cancer, and the tumors’ histologic features, including size and vascular and lymphatic invasion.

The study was conducted in accordance with Resolution nº 466/2012 of the Brazilian National Health Council (NHC).

### DNA extraction

Deparaffinization and extraction of FFPE genomic DNA were performed on three 10-µm thick sections of unstained tumor tissue using the commercial kit ReliaPrep FFPE gDNA Miniprep System (Promega, Madison, WI, USA) according to the manufacturer's instructions. After the extraction process, the purified DNA samples were quantified using fluorometric Qubit dsDNA BR Assay (Invitrogen, Eugene, OR, USA), according to the manufacturer's instructions, and stored at -20 °C until further use.

When the samples did not meet the minimum input DNA amount (1 ng/μL), an attempt was made to re-extract DNA from new sections, if additional slides were available. If the samples did not meet the minimum DNA input after repeat DNA extraction, the sample was considered as "failed" based on insufficient amount and quality of DNA.

### Next-generation sequencing

Genotyping of the target genomic regions (*EGFR* exons 18, 19, 20, and 21; *KRAS* exons 2, 3, and 4; *NRAS* exons 2, 3, and 4; *BRAF* exons 11 and 15; and *PIK3CA* exons 7, 9, and 20) was performed using the platform iSeq 100 Sequencing System (Illumina Inc., San Diego, CA, USA). The library enrichment method was amplicon-based, utilizing the AmpliSeq for Illumina Custom Panel (San Diego, CA, USA) after internal validation and standardization by our partner laboratory, *Laboratório Studart*. The genomic sequences used as reference standards for the analyzed genes were obtained from the National Center for Biotechnology Information (NCBI) nucleotide database: *PIK3CA,* RefSeq: NM_006218; *BRAF*, RefSeq: NM_004333.4; *KRAS*, RefSeq: NM_004985.5; *NRAS*, RefSeq: NM_002524.4; *EGFR*, RefSeq: NM_005228.5. The assay was qualitative and quantitative. The variant allele frequency (VAF) represents the frequency at which a specific genetic variant is observed in a specimen and serves as a parameter for NGS data (25). The laboratory's cutoff value for VAF was set at 5%, and variants with a VAF below 5% were not reported. The average coverage in the regions of interest was 350 times, with 90% of reads in regions of interest having a coverage ≥ 300x, and percentage of reads with a Q value > 30 of > 95%.

Bioinformatic analyses were conducted using the cloud-based platform Varstation (https://varsomics.com/) with a standardized pipeline designed exclusively for the technology and laboratory, following the rules of the Association for Molecular Pathology (AMP) (26-28).

### Statistical analysis

Data processing and analysis were carried out using the software Statistical Package for the Social Sciences (SPSS), version 22 (IBM Corp., Armonk, NY, USA). The analyses were performed using nonparametric tests according to the study's categorical variables. The chi-square test and Fisher's exact test (univariate analyses) were used to evaluate the association between genotyping results and patients’ clinical and pathological characteristics.

## RESULTS

### Clinical and pathological characteristics

The mean age of the study population was 39 years (range 18-88 years), and approximately 24% of the patients were older than 55 years. The female sex was the most prevalent, accounting for 87% of the cases. The tumors’ most frequent histologic subtype was classic PTC, which accounted for 82% of the cases.

The mean tumor size was 2.14 cm (range 0.2-6.6 cm). Overall, 34 tumors were multifocal. Extrathyroidal extension was present in 32% of the cases, and lymph node metastasis in 45% of them (Table 1).

**Table 1:** Clinical and pathological characteristics of the patients included in the study*

Clinical and pathological characteristics		Frequencies
Age at diagnosis (years) – median (range)		39.05 (18-88)
	Patients older than 55 years – n	24
Sex – n (%)		
	Female	87 (87%)
	Male	13 (13%)
Histologic subtype – n (%)		
	Classic PTC	82 (82%)
	Invasive follicular carcinoma	2 (2%)
	Infiltrative follicular variant of PTC	10 (10%)
	Solid variant of PTC	1 (1%)
	Oncocytic variant of PTC	1 (1%)
	Noninvasive encapsulated follicular variant of PTC	1 (1%)
	Cribriform-morular thyroid carcinoma	3 (3%)
Tumor size (cm) – median (range)		2.14 (0.2-6.6)
Tumor multifocality – n (%)		34 (34%)
Extrathyroidal extension – n (%)		32 (32%)
Cervical lymph node metastasis		
	Nx	4 (4%)
	N0	51 (51%)
	N1	45 (45%)

* The study included tumor samples of 100 patients with follicular cell-derived thyroid carcinomas.

Abbreviation: PTC, papillary thyroid carcinoma.

### DNA input and quality assessment according to the age of the FFPE blocks

The FFPE blocks were divided into three groups according to their age: 0-3 years, 4-7 years, and > 7 years. This division was carried out to evaluate whether the age of the paraffin block had any impact on the NGS success rate, as DNA isolated from FFPE tissues is often fragmented, chemically altered, and may have reduced read accuracy, even for short reads.

**Figure 1:**
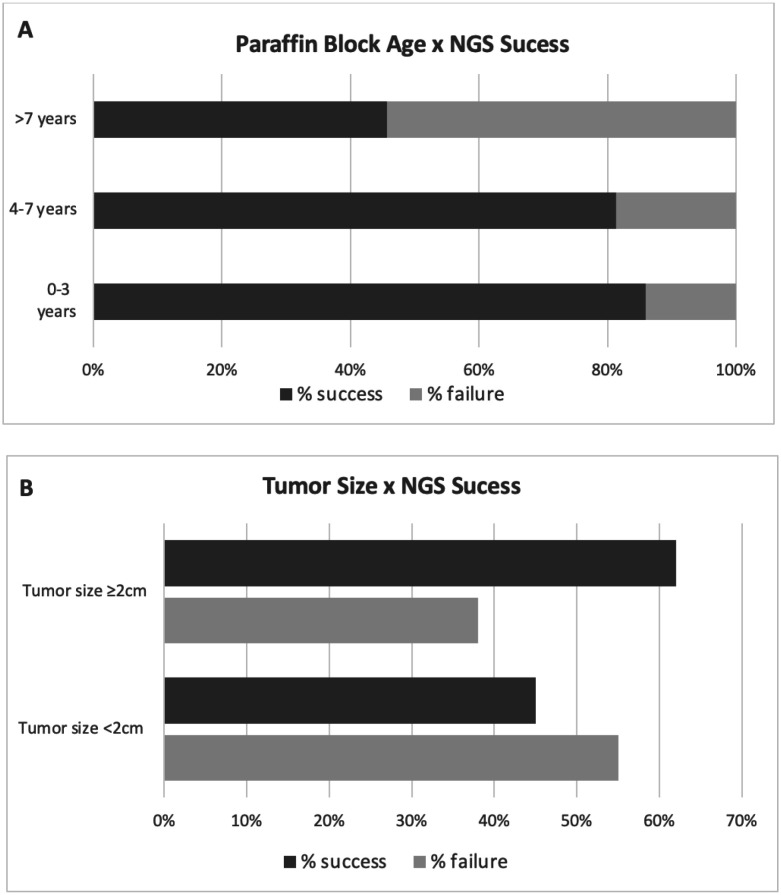
Relationship between (**A**) paraffin block age (0-3, 3-7, and >7 years) and (**B**) tumor size (≥2 and <2 cm) with the success/failure rate of next-generation sequencing (NGS). The success rate was significantly lower in tumors < 2 cm.

As shown in Figure 1A, 86% of the blocks aged 0-3 years were successfully sequenced. However, the sequencing success rate decreased with age, with 81% in blocks aged 4-7 years and 46% in blocks aged >7 years.

We also evaluated the association between tumor size and sequencing (NGS) success rate (Figure 1B). For this analysis, we divided the tumors into two groups, namely, ≥ 2 cm and < 2 cm. The group of tumors < 2 cm had a higher failure rate (55%) than those in the group ≥ 2 cm. The higher sequencing success rate of tumors ≥ 2 cm (62%) may be related to DNA input.

### Multigene panel evaluation

**Figure 2:**
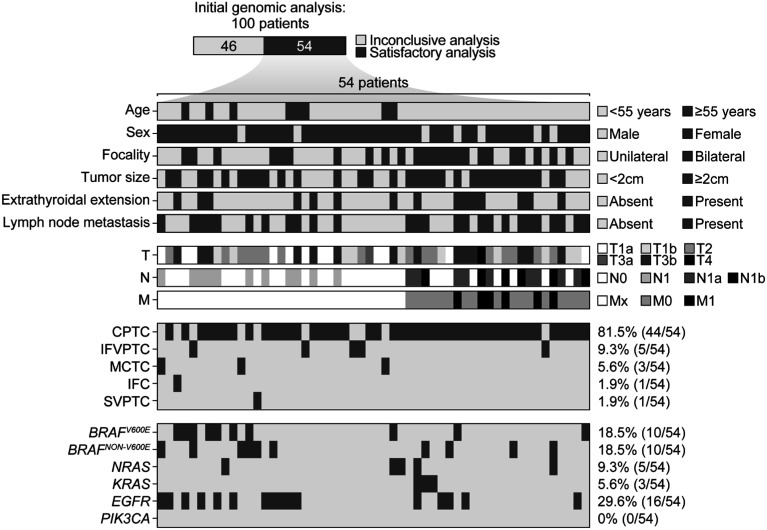
Oncogrid showing the distribution of the oncogenic genetic alterations identified and the patients’ clinical and pathological characteristics. Abbreviations: CPTC, classic papillary thyroid carcinoma; IFC, invasive follicular carcinoma; IFVPTC, infiltrative follicular variant of papillary thyroid carcinoma; SVPTC, solid variant of papillary thyroid carcinoma; OVPTC, oncocytic variant of papillary thyroid carcinoma; EFVPTC, noninvasive encapsulated follicular variant of papillary thyroid carcinoma; MCTC, cribriform-morular thyroid carcinoma.

Of the 100 FFPE samples subjected to the Illumina platform sequencing, 54 had suitable genetic material isolated for an amplicon-based NGS panel analysis. Of these, 23 (42.6%) had no mutations (*i.e.*, wild type) in the five analyzed genes. In the remaining 31 (57.4%) samples, about 44 variants were identified within the target exons, as shown in Figure 2. Interestingly, 12 samples presented more than one genetic variant, with one of the samples harboring three simultaneous genetic variants in different genes. Among the 46 samples with inconclusive results, the block age was > 8 years in 42 (91%).

The most frequent alterations found were genetic variants in exons 11 and 15 of the *BRAF* gene, accounting for approximately 45% of the identified genetic variants (20 out of 44) and present in 18 out of 54 (33%) samples. The *BRAF*^NON-V600E^ variants (*BRAF*^A598V^, *BRAF*^G464E^, *BRAF*^G464R^, *BRAF*^G466E^, *BRAF*^S467L^, *BRAF*^G469E^, *BRAF*^G596D^, and *BRAF*^T599Ifs*10^ deletion), which are less commonly reported, were as frequent as the *BRAF*^V600E^ pathogenic variant (18.5% each; 10 of 54). Two samples harbored a *BRAF*^V600E^ variant and a *BRAF*^NON-V600E^ variant simultaneously.

A total of 16 out of the 54 (29.6%) tumors harbored pathogenic variants in the *EGFR* gene. These included *EGFR*^H850Rfs*26^ deletion (n = 6), *EGFR*^E865K^ (n = 1), *EGFR*^H773Y^ (n = 1), *EGFR*^L792F^ (n = 1), *EGFR*^T725M^ (n = 2), *EGFR*^V729M^ (n = 1), *EGFR*^W817*^ (n = 1), *EGFR*^K754Qfs*7^ (n = 1), *EGFR*^G824Efs*51^ (n = 1), and *EGFR*^L692Hfs*12^ (n = 1). The *RAS* genes (*KRAS* e *NRAS*) harbored eight of the 44 variants identified (18.2%). In seven of the 54 tumor samples (13%), we found *KRAS*^D119N^ (n = 2), *KRAS*^T58I^ (n = 1), *NRAS*^Q61R^ (n = 1), *NRAS*^A146V^ (n = 1), *NRAS*^G12S^ (n = 1), *NRAS*^Q61*^ (n = 1), and *NRAS*^G12D^ (n = 1). One tumor had three simultaneous mutations (*KRAS*^D119N^, *NRAS*^Q61*^, and *EGFR*^H850Rfs*26^); this tumor was a 4-cm classic PTC with extrathyroidal extension and metastasis to a cervical lymph node. We found no genetic alterations in the *PIK3CA* gene.

### Novel *BRAF*^NON-V600E^ variants

The *BRAF*^NON^-^V600E^ variants were found in 10 of the 54 (18.5%) tumor samples. Except for the *BRAF*^A598V^ variant, which has been previously reported (10), 7 of these variants have not been previously described in FCDTCs (*BRAF*^G464E^, *BRAF*^G464R^, *BRAF*^G466E^, *BRAF*^S467L^, *BRAF*^G469E^, *BRAF*^G596D^, and a *BRAF*^T599Ifs*10^ deletion). Regarding the histologic subtypes associated with *BRAF*^NON-V600E^ variants, (A) *BRAF*^G466E^, *BRAF*^G469E^, *BRAF*^G464R^, and *BRAF*^A598V^ were present in classic PTC tumors, (B) *BRAF*^G464E^ and *BRAF*^G596D^ were detected in two cases of cribriform-morular thyroid carcinoma, (C) *BRAF*^T599Ifs*10^ deletion was observed in a solid PTC subtype, and (D) *BRAF*^S467L^ was present in one sample of an infiltrative follicular subtype of PTC and one sample of a classic PTC.

Among the tumors harboring a *BRAF*^NON-V600E^ variant, six had a size > 2 cm and five were metastatic to lymph nodes, while none had extrathyroidal extension (Table 2).

**Table 2:** BRAF^NON-V600^ mutations found in the samples of follicular cell-derived thyroid carcinomas analyzed in the study

Transcript	Coding impact	HGVS coding	HGVS protein	Exon	Histologic subtype	Extrathyroidal extension
NM_004333.6	*Missense*	c.1397G > A	G466E (p. Gly466Glu)	11	CPTC	Absent
NM_004333.6	*Missense*	c.1391G > A	G464E (p. Gly464Glu)	11	CMTC	Absent
NM_004333.6	*Missense*	c.1390G > A	G464R (p. Gly464Arg)	11	CPTC	Absent
NM_004333.6	*Missense*	c.1400C > T	S467L (p. Ser467Leu)	11	CPTC IFVPTC	Absent
NM_004333.6	*Missense*	c.1787G > A	G596D (p. Gly596Asp)	15	CMTC	Absent
NM_004333.6	*Frameshift*	c.1796_1803del	T599Ifs*10 (p. Thr599IlefsTer10)	15	SVPTC	Absent
NM_004333.6	*Missense*	c.1406G > A	G469E (p. Gly469Glu)	11	CPTC	Absent

Abbreviations: CMTC, cribriform-morular thyroid carcinoma; CPTC, classic papillary thyroid carcinoma; HGVS, Human Genome Variation Society; IFVPTC, infiltrative follicular variant of papillary thyroid carcinoma; SVPTC, solid variant of papillary thyroid carcinoma.

**Figure 3:**
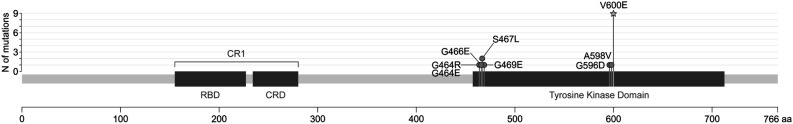
*BRAF* domain structure and location of the hotspot mutations found in the study. Abbreviations: RBD, RAS-binding domain; CRD, cysteine-rich domain.

Of the eight *BRAF*^NON-V600E^ variants found, five were in exon 11, and three were in exon 15. Our findings are summarized in a schematic diagram of the BRAF protein showing the N-terminal CR1 region, which contains the RAS-binding domain (RBD) and the cysteine-rich domain (CRD), and the C-terminal CR3 region, which contains the serine/threonine kinase domain (29,30). No variants were found in the RBD or CRD domains (Figure 3).

## DISCUSSION

The knowledge of the mutational status of genes involved in the MAPK and PI3K-AKT pathways is key to a proper understanding of the behavior of FCDTCs. Patients with the same histologic subtypes of FCDTC can differ substantially in terms of disease progression, severity, and prognosis, depending on their molecular classification (8,9,31,32). The molecular understanding of FCDTCs opens up possibilities for targeted therapies with selective kinase inhibitors for those patients who have actionable mutations and do not respond to conventional treatment due to the tumor's increased aggressiveness and progression (33).

In the literature, the frequency of the *BRAF*^V600E^ variant in adults with PTC ranges from 27% to 83% (8,11,13,15,16). In the present study, the frequency of *BRAF* mutations was 33% (18 out of 54), but the frequency of the *BRAF*^V600E^ variant, in particular, was 18.5% (10 out of 54). This low frequency may be related to the reduced number of cases that were suitable for NGS analysis. In this study, *BRAF*^V600E^ mutations were predominantly found in tumors with a classic PTC histologic subtype (eight cases), but they were also observed in one case of invasive follicular carcinoma and one case of infiltrative follicular variant of PTC.

The present study identified various *BRAF*^NON-V600E^ variants. Among these variants, only *BRAF*^A598V^ has been previously described in a case of FCDTC (5). Other studies (34,35) have reported a deletion at T599, but not in the same codon described in our study. Thus, our study is the first to report the *BRAF*^G464E^, *BRAF*^G464R^, *BRAF*^G466E^, *BRAF*^S467L^, *BRAF*^G469E^, *BRAF*^G596D^, and the *BRAF*^T599Ifs*10^ deletion variants in FCDTC. Of note, these variants have been described in other types of malignancies, including skin/melanoma (*BRAF*^G464R^, *BRAF*^G466E^, *BRAF*^S467L^, *BRAF*^G469E^, and *BRAF*^G596D^), lung (*BRAF*^G464E^, *BRAF*^G464R^, *BRAF*^G466E^, and *BRAF*^S467L^), and colorectal (*BRAF*^G464E^, *BRAF*^G466E^, *BRAF*^S467L^, *BRAF*^G469E^, and *BRAF*^G596D^) cancers and in other types of cancer (36-52). These novel findings may be explained by the scarcity of thyroid cancer studies analyzing the exon 11 of the *BRAF* gene, as most studies focus on exon 15, where the V600E variant is located.

In melanoma (53) and non-small cell lung cancer (54), *BRAF*^NON-V600E^ mutations have been associated with increased disease aggressiveness compared with the *BRAF*^V600E^ pathogenic variant. However, in colorectal cancer (55), patients with *BRAF*^NON-V600E^ variants have shown significantly longer survival compared with those with *BRAF*^V600E^. In the present study, *BRAF*^NON-V600E^ variants were present in six cases of classic PTC, two cases of cribriform-morular thyroid carcinoma, one case of solid variant PTC, and one case of infiltrative follicular variant of PTC.

The presence of extrathyroidal extension is an important prognostic factor in thyroid cancer, as pointed out by Liu and cols. (2016) (56). The risk of extrathyroidal extension increased by 2.04 times in tumors with a positive *BRAF*^V600E^ pathogenic variant compared with wild-type cases. In the present study, extrathyroidal extension was present in 40% of the tumors harboring a *BRAF*^V600E^ mutation but was absent in tumors with *BRAF*^NON-V600E^ variants (p = 0.046). Therefore, our results suggest that *BRAF*^NON-V600E^ variants do not exhibit the same risk association with extrathyroidal extension as described for the classic *BRAF*^V600E^ variant.

Regarding the variants in the *RAS* gene, they are more associated with follicular thyroid cancer and FCDTC and are less frequent in classic PTC (10%-20%) (8,9,11). In the present study, the prevalence of *RAS* variants in PTC was 13% (7 out of 54), which is consistent with data from the literature. The variants found in the *NRAS* isoform were more prominent, representing 9.2% (5 of 54), while variants in the *KRAS* isoform were found in 5.5% (3 of 54) of cases. These findings are also aligned with results from previous studies, which have reported a higher frequency of *NRAS* than *KRAS* variants in FCDTCs (8,9,11). In contrast to previous studies (23,57), ours found that *RAS* mutations coexisted with *BRAF* mutations in three of our classic PTC cases, specifically, one with concomitant *BRAF*^V600E^/*NRAS*^A146V^ and two with *BRAF*^NON-V600E^/*KRAS*^D119N^ and *NRAS*^G12D^. These findings have also been reported in another study (58) of four PTC tumors harboring simultaneous *BRAF*^V600E^ and *KRAS*^G12D^ mutations, which were associated with disease progression. One explanation for this finding is that few studies track all *RAS* genes in FCDTCs with a *BRAF*^V600E^ mutation (58). Although a rare event, the combination of *BRAF*^V600E^ and *RAS* mutations has already been described in other types of cancer, including colorectal carcinoma (59), melanoma (60), and gastric cancer (61).

One of the tumors in the present study had concurrent *KRAS*^D119N^, *NRAS*^Q61*^, and *EGFR*^H850Rfs*26^ mutations. This finding was associated with increased disease aggressiveness and worse prognosis due to the tumor's size (4 cm) and presence of extrathyroidal extension and lymph node metastasis. Some studies (62-64) have correlated *RAS* gene mutations with a poorer prognosis, suggesting that the detection of *RAS* gene mutations may be clinically relevant for diagnosis and risk stratification. However, this topic is still controversial. In contrast, other studies (2,65) have found that *RAS* variants alone offer little utility for the diagnosis of malignancy, as they can also be found in benign follicular adenomas and are not necessarily involved in thyroid tumor progression, requiring additional genetic alterations. Despite the limitation of the small number of cases with *RAS* variants in our study, we still believe that the inclusion of *RAS* in multigene analysis panels is important, as it may yield prognostic information, especially when associated with other mutations.

Mutations in the *EGFR* gene have been poorly studied in FCDTC. A study by Masago and col. (66) (2009) found *EGFR* mutations in 30.4% of patients with PTC. Our study found a close prevalence (29.6%; 16 of 54) but with different variants. No *PIK3CA* variants were found in the FCDTCs analyzed in the present study; this finding is consistent with the literature (13,33), in which a low prevalence of these mutations has been reported in PTC and follicular thyroid cancer.

The limitations of our study include the high failure rate of the NGS analysis (46%), which resulted in a reduced number of samples with satisfactory results. Many factors during the FFPE fixation process may affect DNA and RNA quality in both the pre-fixation and post-fixation steps. Other factors affecting the quality of DNA include cross-linking, delayed fixation, formalin pH, and fixation time, which in turn affect the target coverage depth in NGS. Most of the older blocks were likely not fixed in a buffered formalin solution (thus, were maintained in acidic pH), which may have caused DNA degradation. Some studies (67-69) have found DNA degradation in FFPE samples due to fragmentation and chemical modification from prolonged formalin fixation time, acidic pH conditions, and paraffin embedding, resulting in failed NGS analysis, particularly in FFPE tumor samples older than 4-6 years. In our study, 91% of the failures occurred in samples that had been collected more than 8 years before, which is consistent with the findings in the cited studies.

In conclusion, this study found new variants in the *BRAF* gene (*BRAF*^G464E^, *BRAF*^G464R^, *BRAF*^G466E^, *BRAF*^S467L^, *BRAF*^G469E^, *BRAF*^G596D^, and *BRAF*^T599Ifs*10^ deletion) that had not been previously described in FCDTCs. The *BRAF*^NON-V600E^ variants were particularly frequent in this study but did not show a significant association with clinical and pathological parameters (*e.g.*, disease aggressiveness) and were associated with a lower risk of extrathyroidal extension. However, the frequency of these alterations in FCDTC and their long-term impact are concerning, and further studies are needed to better classify and correlate them with clinical and pathological features.

## Data availability:

the datasets analyzed in the current study are available in the Supplementary Table.

## Supplementary Table Datasets analyzed in the study

**Table:**

Sample ID	NGS Results	VAF (%)
1	*NM_004333.6(BRAF):c.1787G > A (p. Gly596Asp) NM_005228.5 (EGFR):c.2593G > A:p.(Glu865Lys)*	10.6 17.8
2	*NM_005228 (EGFR):c.2549_2610del:p.(His850Argfs*26)*	87.5
3	*NM_004333.6 (BRAF):c.1799T > A:p.(Val600Glu)*	11.7
4	*NM_004333.6(BRAF):c.1799T > A:p.(Val600Glu) NM_005228 (EGFR):c.2549_2610del:p.(His850Argfs*26)*	26.1 8.1
5	*NM_004333.6 (BRAF):exon15:c.1799T > A:p.(Val600Glu) NM_004333 (BRAF):c.1400C > T:p.(Ser467Leu)*	19.4 30.8
6	*NM_005228.5(EGFR):c.2317C > T:p.(His773Tyr)*	17.9
7	*NM_004333.6 (BRAF):c.1799T > A:p.(Val600Glu)*	30.6
8	*NM_004333.6 (BRAF):c.1799_1806del:p.(Val600Alafs*9) NM_005228.5(EGFR):c.2374C > T:p.(Leu792Phe)*	4.2 13
9	*NM_002524.5 (NRAS):c.182A > G:p.(Gln61Arg)*	33.7
10	*NM_004333.6 (BRAF):c.1799T > A:p.(Val600Glu) NM_005228 (EGFR):c.2549_2610del:p.(His850Argfs*26)*	22.4 51.1
11	*NM_004333.6(BRAF):c.1391G > A:p.(Gly464Glu)*	7.4
12	*NM_004333.6(BRAF):c.1799T > A:p.(Val600Glu) NM_004333.6(BRAF):c.1397G > A:p.(Gly466Glu)*	17.5 10.1
13	*NM_004333.6 (BRAF):c.1796_1803del:p.(Thr599Ilefs*10)*	39.4
14	*NM_005228.5(EGFR):c.2174C > T:p.(Thr725Met)*	14.8
15	*NM_004333.6(BRAF):c.1793C > T:p.(Ala598Val) NM_005228(EGFR):c.2549_2610del:p.(His850Argfs*26)*	18.8 23.8
16	*NM_005228(EGFR):c.2305G > A:p.(Val769Met)*	17
17	*NM_005228.5(EGFR):c.2450G > A:p.(Trp817Ter)*	37.1
18	*NM_005228.5(EGFR):c.2259_2265del:p.(Lys754Glnfs*7)*	50
19	*Wild type*	-
20	*Wild type*	-
21	*Wild type*	-
22	*Wild type*	-
23	*Wild type*	-
24	*Wild type*	-
25	*Wild type*	-
26	*Wild type*	-
27	*Wild type*	-
28	*Wild type*	-
29	*Wild type*	-
30	*NM_004333.6(BRAF):c.1799T > A:p.(Val600Glu) NM_002524.5(NRAS):c.437C > T:p.(Ala146Val)*	21.1 11.7
31	*NM_002524.5(NRAS):c.34G > A:p.(Gly12Ser)*	35.5
32	*Wild type*	-
33	*NM_002524.5 (NRAS):c.181C > T:p.(Gln61Ter) NM_004985.5(KRAS):c.355G > A:p.(Asp119Asn) NM_005228.5 (EGFR):c.2549_2610del:p.(His850Argfs*26)*	29.0 4.0 92.0
34	*NM_004333(BRAF):c.1390G > A:p.(Gly464Arg) NM_004985.5(KRAS):c.355G > A:p.(Asp119Asn)*	7.0 7.0
35	*NM_004985.5(KRAS):c.173C > T:p.(Thr58Ile)*	21
36	*NM_005228.5(EGFR):c.2174C > T:p.(Thr725Met)*	6
37	*NM_004333 (BRAF):c.1400C > T:p.(Ser467Leu) NM_005228.5(EGFR):c.2470_2534del:p.(Gly824Glufs*51)*	6.0 60.0
38	*NM_004333.6:(BRAF):c.1799T > A:p.(Val600Glu)*	32
39	*NM_005228.5(EGFR):c.2073_2076del:p.(Leu692Hisfs*12)*	59
40	*Wild type*	-
41	*Wild type*	-
42	*Wild type*	-
43	*Wild type*	-
44	*Wild type*	-
45	*NM_004333.6(BRAF):c.1400C > T:p.(Ser467Leu)*	18
46	*Wild type*	-
47	*Wild type*	-
48	*Wild type*	-
49	*Wild type*	-
50	*NM_004333.6 (BRAF):c.1406G > A:p.(Gly469Glu) NM_002524(NRAS):c.35G > A:p.(Gly12Asp)*	5.0 5.0
51	*Wild type*	-
52	*Wild type*	-
53	*NM_005228.5 (EGFR):c.2549_2610del:p.(His850Argfs*26)*	6
54	*NM_004333.6(BRAF):c.1799T > A:p.(Val600Glu)*	6

Abbreviations: ID, identification; NGS, next-generation sequencing; VAF, variant allele frequency.
